# Relationship between HIV integrase polymorphisms and integrase inhibitor susceptibility: An *in silico* analysis

**DOI:** 10.1016/j.heliyon.2018.e00956

**Published:** 2018-12-01

**Authors:** Hotma Martogi Lorensi Hutapea, Yustinus Maladan, Widodo

**Affiliations:** a Institute of Health Research and Development Papua, Ministry of Health, Indonesia; b Biology Department, Faculty of Mathematics and Natural Sciences, Universitas Brawijaya, Malang, Indonesia

**Keywords:** Molecular biology, Bioinformatics, Biochemistry, Structural biology

## Abstract

Integrase (IN) plays an essential role in HIV-1 replication, by mediating integration of the viral genome into the host cell genome. IN is a potential target of antiretroviral (ARV) therapeutic drugs such as ALLINI, Raltegravir (RAL), and Elvitegravir (EVG). The effect of IN polymorphisms on its structure and binding affinity to the integrase inhibitors (INIs) is not well understood. The goal of this study was to examine the effect of IN polymorphisms on its tertiary structure and binding affinities to INIs using computational approaches. HIV genomes were isolated from patient blood and the IN gene was sequenced to identify polymorphisms. Protein structures were derived using FoldX and the binding affinity of IN for ALLINI, RAL, and EVG was evaluated using a molecular docking method. The binding affinities of ALLINI and EVG for wild-type IN were lower as compared to an IN variant; in contrast, the binding affinity of RAL for the IN variant was lower as compared to wild-type IN. These results suggested that IN variant interacts with ALLINI and EVG more efficiently as compared to the wildtype, which may not cause resistent to the drugs. *In vitro* and *in vivo* studies should be done to validate the findings of this study.

## 1 Introduction

Human Immunodeficiency Virus type 1 (HIV-1) integrase (IN) is an enzyme responsible for the integration of the double-stranded DNA form of the HIV-1 genome into the genome of infected cells. IN is composed of a central core domain that contains a conserved domain, D-35-E motif. Mutation of any of the residues in this motif diminishes all catalytic activity of the protein [1].

Integration of a cDNA copy of the viral genome into the infected cell's genome is an obligatory step in the replication of all retroviruses. HIV-1 replication is a multistep process, which includes: 1) assembly of a stable complex between IN and specific viral DNA sequences at the end of the HIV-1 Long Terminal Repeat (LTR), 2) cleavage of the viral CA dinucleotide (3′ processing), 3) preintegration complex translocation, 4) strand transfer, and 5) DNA gap repair and ligation. Any of these steps can be considered as a potential target of inhibitory drugs [2].

IN inhibitors (INIs) block IN activity, which prevents integration of the viral double-stranded DNA into the host cell's genomic DNA. Raltegravir (RAL) was the first INI approved for clinical use in both treatment-naive and treatment-experienced patients [3]. RAL is also useful for treating patients, which are infected by antiretroviral-resistant HIV-1 strain [4]. Elvitegravir (EVG) is another ARV that was approved by the FDA for clinical treatment of patients. Both RAL and EVG inhibit IN activity by forming a complex with viral DNA, which hinders the process of viral genome integration [5]. Another type of INI is the Allosteric Integrase Inhibitor (ALLINI) class that works by inhibiting binding interactions between IN and Lens Epithelium Derived Growth Factor (LEDGF/p75), which reduces IN catalytic activity [6]. ALLINIs (also referred to as LEDGINs, noncatalytic site integrase inhibitors (NCINIs), or multimodal inhibitors) are highly active against HIV replication in cell culture [7].

HIV-1 is characterized by extensive genetic heterogeneity resulting from the absence of reverse transcriptase (RT) proofreading activity, which leads to high genetic variability and rapid evolution of HIV-1. Genetic heterogeneity originates from the high mutation rate of RT [8]. Genetic mutations ultimately lead to drug resistance, yet little is known about IN polymorphisms, whereas a study on polymorphisms of HIV-1 RT and Protease has been conducted [9]. In this study, polymorphisms of HIV IN (i.e., IN variants) from one viral isolate were analyzed *in silico*.

## 2 Methods

### 2.1 Amplification of HIV-1 integrase

A blood from an HIV-1 infected patient without INI treatment was obtained from Biobank Management in the Institute of Health Research and Development Papua, Ministry of Health, Indonesia that served as the source for the RNA genome of HIV-1. A 546 bp fragment of the IN gene was reverse transcribed and amplified using a one-step RT-PCR with primers pintDOF: 5′–Tgg AgA gCA Atg gCT AgT gA–3′ and pintDOR: 5′–CTA TTC TTT CCC CTg CAC TgT–3′. RT-PCR thermal cycling conditions consisted of: 50 °C: 30 minutes; 95 °C: 2 minutes; 35 cycles of: 0.5 minute at 95 °C, 0.5 minute at 47 °C, 1 minute at 72 °C; with a final extension of 72 °C for 7 minutes.

### 2.2 Sequencing

PCR products of the IN gene were purified by ExoSap IT (Thermo Fisher Scientific). Purified PCR products were sequenced using protocol of Genetic Anlyzer 3500 (Thermo Fisher Scientific). The sequencing PCR was done as follow; mixture of 2.5X BigDye Terminator v3.1(4 μL), BigDye Terminator buffer v1.1/v3.1 5X (4 μL), 1 μL DNA, and nuclease-free water (7 μL); then were conducted a sequencing-PCR with condition 1 minute at 96 °C, followed by 25 cycles of 10 seconds at 96 °C, 5 seconds at 50 °C, and 4 minutes at 60 °C. The sequencing-PCR products were purified with XTerminator Solution and SAM solution in rasio 10:45 (premix). The tube containing the premix and PCR product was vortexed for 30 minutes prior to homogenization. Thereafter, the tube was centrifuged at 1,000 ×g for 1 minute. The supernatant was analyzed with a 3500 Genetic Analyzer. The sequencing results were compared with wildtype of HIV integrase on the *Stanford HIVdb mutation interpretation* database to identify the presence of mutations in the IN gene.

### 2.3 Three-dimensional structural modeling of mutant HIV-1 integrase

FoldX software [10] was used for structural modeling of IN variant. The three-dimensional structure of the IN variant was derived using the multiple mutations method based on published HIV integrase in the Protein Data Bank (PDB), PDB ID:5HOT. Energy stability, site chain orientation, and surface molecules were analyzed using Yasara software, which has been widely used in protein modeling studies [11, 12, 13]. Additional analysis of hydrophobicity was performed with Discovery Studio software (http://accelrys.com).

### 2.4 Binding affinity of mutant HIV-1 integrase with integrase inhibitor drugs

Docking analysis was performed with AutoDock Vina [14], which is integrated into the PyRx application (https://pyrx.sourceforge.io/) for analysis of docking results [15]. The docking Grid was set on the ALLINI binding site; this strategy also for identify whether EVG and RAL might also work in same function with ALLINI. Docking results were visualized using Discovery Studio software. The 3D structures of INIs (ALLINI, EVG, and RAL) were downloaded from the drug bank database, available at www.drugbank.ca.

## 3 Results and discussion

**Fig. 1:**
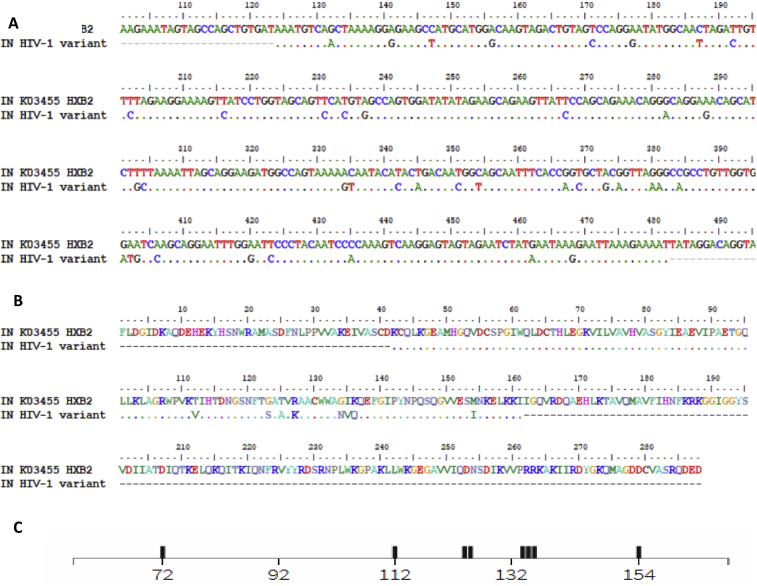
Polymorphisms in the IN gene. (A) Alignment of IN gene sequences of the CRF01_AE isolate and the HXB2 reference strain (GenBank accession number K03455); (B) Alignment of amino acid of IN protein to IN gene translation from the patient with sequence reference HXB2 (accession number: K03455); (C) The following polymorphisms, based on the *Stanford HIVdb mutation interpretation* database, were identified in the IN gene of the CRF01_AE isolate: I72V, T112V, T124A, T125A, G134N, I135V, K136Q, and M154I.

The IN gene sequence corresponding to the Jayapura isolate (CRF01_AE) was aligned to the wild-type reference sequence (GenBank accession number K03455; reference strain HXB2). The alignment revealed several polymorphisms in the IN gene of CRF01_AE (Fig. 1A). Based on the *Stanford HIVdb mutation interpretation* database, the following polymorphisms were identified: I72V, T112V, T124A, T125A, G134N, I135V, K136Q and M154I (Fig. 1B). EVG-resistance related polymorphisms Q148H and N155H [15] were not observed in the CRF01_AE.

**Fig. 2:**
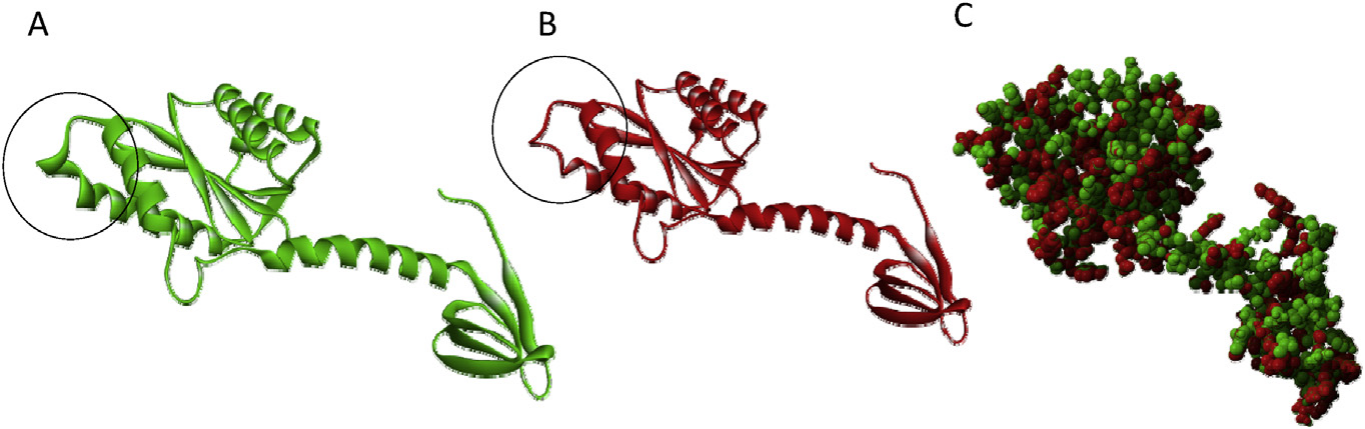
Three-dimensional structure of IN. (A) Wild-type IN contains a longer helix domain (circled) as compared to the the mutant IN (B). This structural difference was due to amino acid polymorphisms near the INI binding pocket, as seen in the overlay of the wild-type (green) and variant (red) structures (C).

The three-dimensional structure of the variant IN protein, derived using FoldX software, revealed that the alteration was caused by amino acid changes [10]. Structural modeling revealed that wild-type IN contains a longer helix domain as compared to the variant IN (Fig. 2). Minimal, if any, differences of protein stability were detected between the wild-type and variant IN (Table 1).

**Table 1:** Stability of wild-type and variant IN.

No.	Criteria	Wild-type IN	Variant IN
1	Energy after repair & minimization	−11,412.42 kJ/mol	−11,901.82 kJ/mol
2	Stability after repair & minimization	188.99 kcal/mol	189.02 kcal/mol

**Fig. 3:**
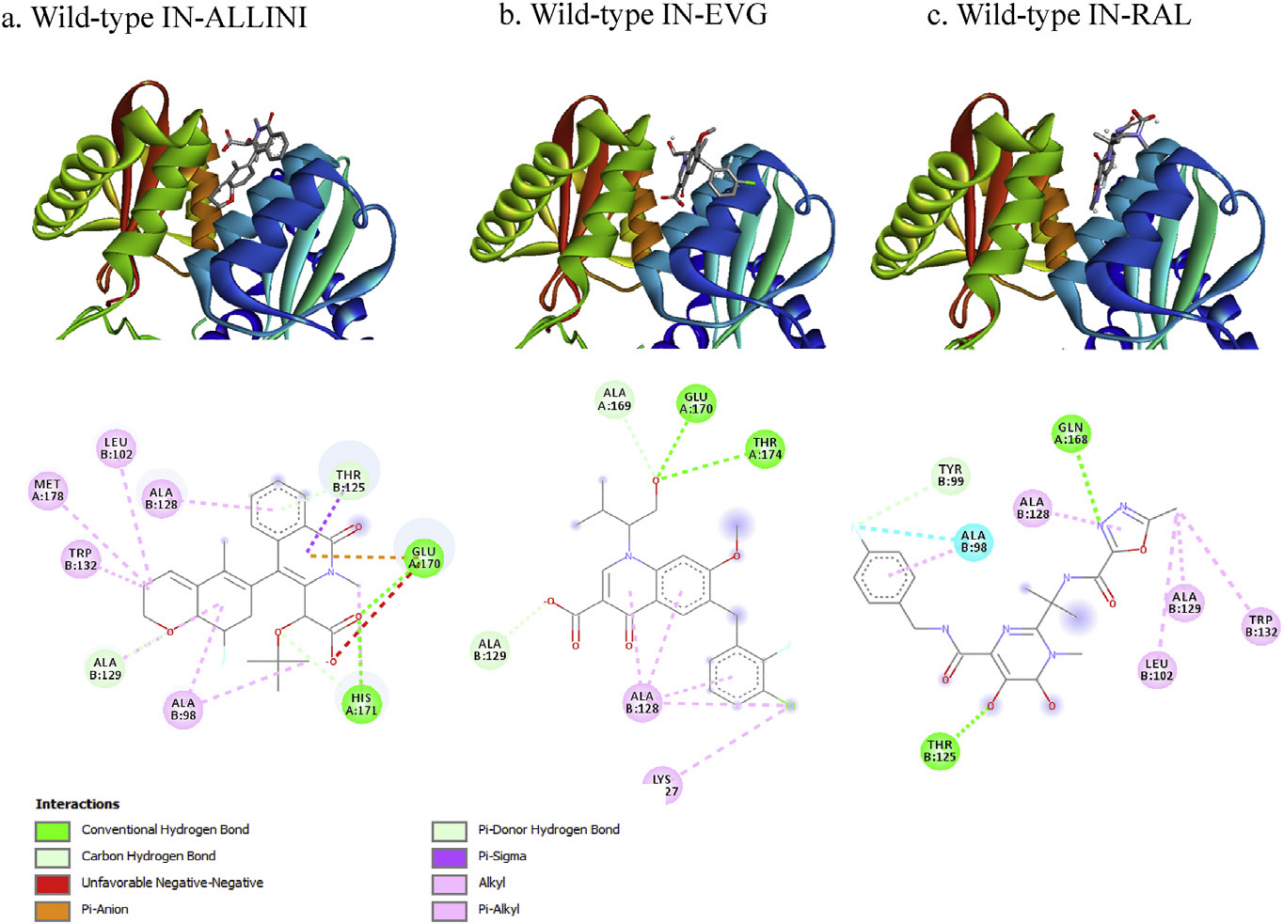
INI interactions with the wild-type IN binding pocket. a) Interaction of wild-type IN with ALLINIs, which interact with IN via hydrogen bonding with residues Glu170 (and His171. b) Interaction between EVG and wild-type IN, EVG interacts with IN via hydrogen bonding with residues Glu170 and Thr174. c) Interactions between RAL and wild-type IN, RAL interacts with IN via hydrogen bonding with residues Thr125 and Gln168.

**Fig. 4:**
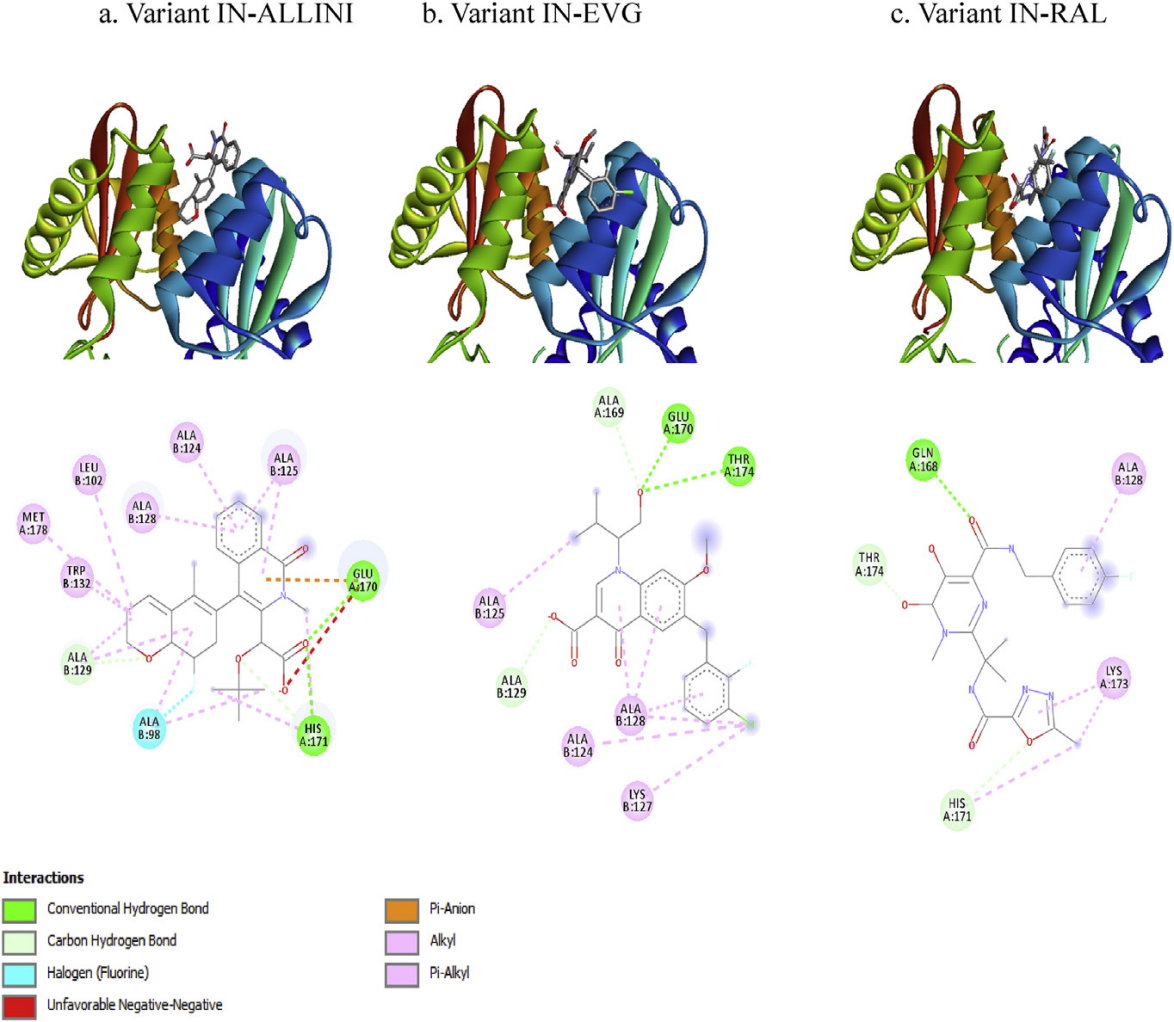
INI interactions with variant IN binding pocket. a) Interaction of variant IN with ALLINI, which hydrogen bonds with Glu170 and His171. b) interaction of EVG with variant IN, EVG interacts via hydrogen bonding with residues Glu170 and Thr174. c) RAL interacts with variant IN through hydrogen bonding with residues Gln168 and His171.

Amino acid polymorphisms in the variant IN were predicted to slightly alter the structure, stability, and hydrophobicity of the protein, which changed the binding properties of the INIs (Fig. 3). The interaction of INIs with IN was mediated via hydrogen bonding, carbon-hydrogen bonding, unfavorable negative-negative, pi-anion, pi-donor hydrogen bonding, pi-sigma, alkyl, and pi-alkyl (Figs. 3 and 4). The variant IN may have a stronger binding affinity for ALLINIs and EVG, but not for RAL. However, changes in binding affinities were only minimally altered, although the binding affinity of ALLINI and EVG for the variant IN appeared to be stronger as compared to wild-type IN (Table 2).

**Table 2:** INI and IN binding affinities.

Integrase inhibitor	Binding affinity (Kcal/mol)	
	Wild-type	Variant
ALLINIs	−7.6	−8.3
EVG	−7.2	−8.1
RAL	−7.8	−7.2

The IN gene of the HIV isolate from Papua (CRF01_AE isolate) contains genetic polymorphisms (Fig. 1A) that encode for variant amino acids in the IN protein, based on the *Stanford HIVdb mutation interpretation* database (Fig. 1B). These variations slightly changed the three-dimensional structure of IN. Wild-type IN contains a longer helix domain as compared to variant IN (Fig. 2). However, this structural change had a minimal to no effect on IN stability (Table 1). This result may be due to the fact that the amino acid substitutions are not located in the conserved central core domain of IN (Asp64, Asp116, and Glu152) [16]. Molecular docking analysis indicated that the ALLINIs, EVG, and RAL did not much change the binding affinity to the wildtype or variant IN (Table 2). These three INIs can bind in the wild-type and variant IN(Fig. 3). IN residues 125, 128, 170, 171, and 173 are positioned to make contact with INIs; substitutions at these amino acid positions likely disrupt the inhibitor-mediated interface directly [7].

IN resistance to ALLINI is dependent on the A128T polymorphism [17], which was not observed in this study. ALLINI bound to residues Glu170 and His171 of both the wild-type and variant IN. ALLINI also interacts with ALA98 and A125 of variant IN via a halogen bond and a pi-alkyl interaction, respectively (Fig. 4a). A study conducted by Lu et al. demonstrated that halogen bonding plays an important role in inhibitor recognition and binding to the targeted protein [18]. The unique chemical characteristics of halogens are beneficial to the design of protein inhibitors and drugs [19]. The ALLINIs showed relatively higher binding affinity to the variant IN as compared to wild-type.

The IN polymorphisms T66I/A, V72I, F121Y, T125K, G140C, S147G, Q148H, V151I, S153Y, M154I, and S230R mediate resistance to EVG [20]. The IN variant in this study contained M154I; however, according to the docking analysis, EVG bound more strongly to the IN variant as compared to wild-type. The binding affinity between EVG and variant IN was −8.1 kcal/mol, but −7.2 kcal/mol with the wild-type IN (Table 2). Residues Glu170 and Thr174 of both wild-type and variant form hydrogen bonds with EVG. However residues Ala124 and Ala125 of the IN variant, but not wild-type, formed pi-alkyl interactions with EVG.

The polymorphisms Q148H/K/R, N155H, and Y143H of IN were associated with the resistance of HIV-1 to RAL [21]. These polymorphisms were not found in this study; however the binding affinity between RAL and the variant IN was decreased as compared to the wild-type IN, which was due to the loss of hydrogen bonding between RAL with Thr125 in variant IN and loss of several other interactions between RAL and Leu102, Ala128, Ala129, and Trp132.

In summary, the study showed that ALLINI and EVG increased binding affinity to the variant-IN compared to the wild-type of integrase. The variant IN had a lower affinity for RAL as compared to wild-type IN. This suggests that the variant IN may be less susceptible to the development of drug resistance mutations. However, additional research should be done to further evaluate these results.

## Declarations

### Author contribution statement

Hotma Martogi Lorensi Hutapea: Conceived and designed the experiments; Performed the experiments; Analyzed and interpreted the data; Contributed reagents, materials, analysis tools or data; Wrote the paper.

Yustinus Maladan, Widodo: Analyzed and interpreted the data; Contributed materials, analysis tools or data; Wrote the paper.

### Funding statement

This research was funded by Ministry of Health, Republic of Indonesia.

### Competing interest statement

The authors declare no conflict of interest.

### Additional information

No additional information is available for this paper.
